# Deep-Sequencing Protocols Influence the Results Obtained in Small-RNA Sequencing

**DOI:** 10.1371/journal.pone.0032724

**Published:** 2012-02-27

**Authors:** Joern Toedling, Nicolas Servant, Constance Ciaudo, Laurent Farinelli, Olivier Voinnet, Edith Heard, Emmanuel Barillot

**Affiliations:** 1 Institut Curie, Paris, France; 2 INSERM U900, Paris, France; 3 Mines ParisTech, Fontainebleau, France; 4 CNRS UMR3215, Paris, France; 5 INSERM U934, Paris, France; 6 Department of Biology, Swiss Federal Institute of Technology Zürich, Zürich, Switzerland; 7 Fasteris, Plan-les-Ouates, Switzerland; 8 Institut de Biologie Moléculaire des Plantes, CNRS UPR2357 – Université Louis Pasteur, Strasbourg, France; Inserm U869, France

## Abstract

Second-generation sequencing is a powerful method for identifying and quantifying small-RNA components of cells. However, little attention has been paid to the effects of the choice of sequencing platform and library preparation protocol on the results obtained. We present a thorough comparison of small-RNA sequencing libraries generated from the same embryonic stem cell lines, using different sequencing platforms, which represent the three major second-generation sequencing technologies, and protocols. We have analysed and compared the expression of microRNAs, as well as populations of small RNAs derived from repetitive elements. Despite the fact that different libraries display a good correlation between sequencing platforms, qualitative and quantitative variations in the results were found, depending on the protocol used. Thus, when comparing libraries from different biological samples, it is strongly recommended to use the same sequencing platform and protocol in order to ensure the biological relevance of the comparisons.

## Introduction

Over recent years, second-generation sequencing (NGS) has established itself as the method of choice for efficiently determining the nucleotide sequences of large collections of RNA/DNA molecules. At present, three different technologies are most commonly used for performing large-scale sequencing: 454 (Roche), Solexa (Illumina) and SOLiD (Life Technologies) (see [1] for a review).

NGS technologies provide a powerful approach for the analysis of small (<50 nt), non-coding RNAs (ncRNAs), enabling quantitative measurements of previously annotated ncRNA populations, as well as the identification of novel ncRNAs [2]. While standards for these experiments are still lacking, the preparation of small-RNA sequencing (sRNA-seq) as well as the downstream analysis can have strong impacts on the biological interpretations and conclusions [3]. Comparisons of the results obtained between technologies remain sparse however. Differences have previously been reported in small RNA expression measurements obtained from the same biological sample using three different sequencing technologies (454, ABI SOLiD and traditional capillary dideoxy sequencing platforms) and qPCR [4] [5], as well as using different RNA ligases [6]. In this report, we have investigated several small-RNA sequencing libraries generated using different sequencing technologies (Roche-454, Illumina-Solexa, ABI-SOLiD), protocols (Homemade, two Illumina, the Life Technology SREK and STaR-Seq kits) and adaptors (IDT [7] et Illumina) for Solexa sequencing.

We present a comparison of sRNA-seq data from two mouse embryonic stem (ES) cell lines generated using the three major NGS platforms (454, Solexa, SOLiD). The libraries consist of short RNAs, approximately 19–30 nt in length, from two mouse ES cell lines, E14 male and PGK female, in the undifferentiated state. All of the libraries investigated are listed in Table 1.

**Table 1:** Description of the libraries investigated.

SampleID	CellType	Technology	Year	Barcode/index	Comment	# reads
ES_XY_454	E14 XY	454	2008	barcode	Ciaudo et al. (2009)	95203
ES_XY_Solexa_Illu	E14 XY	Solexa	2010	none	GAIIx/Illumina 3′ adapter	28014973
ES_XY_Solexa_i_IDT	E14 XY	Solexa	2010	Index	HiSeq2000/IDT 3′ adapter	8375905
ES_XY_Solexa_IDT	E14 XY	Solexa	2010	none	GAIIx/IDT 3′ adapter	31316082
ES_XY_SOLID_v3	E14 XY	SOLiD	2010	none	v3+/SREK kit	32685742
ES_XY_SOLID_v4	E14 XY	SOLiD	2010	barcode	v4/STaR-Seq kit	2685423
ES_XX_454	PGK XX	454	2008	barcode	Ciaudo et al. (2009)	57497
ES_XX_Solexa_i_IDT	PGK XX	Solexa	2009	Index	HiSeq2000/IDT 3′ adapter	10262556
ES_XX_SOLID_v3	PGK XX	SOLiD	2010	none	v3+/SREK kit	32974547
ES_XX_SOLID_v4	PGK XX	SOLiD	2010	barcode	v4/STaR-Seq kit	2714593

The ten samples differ in size, in the employed sequencing technology, in the version of the machine that they were generated with and whether a barcode or index had been used for parallel sequencing with other libraries.

In addition, we have also compared the effect of indexing samples for multiplexing during Solexa library preparation, as it has been previously suggested that barcoding could have an impact on deep-sequencing results [8]. While barcodes are usually attached to the RNA adaptor sequence and integrated at the ligation step, indexes are introduced with the PCR primers during the amplification step (Figure S1 adapted from Pfeffer et al. [9]).

## Results

Ten small-RNA libraries had been sequenced using different technologies and protocols. The different steps and results of the bioinformatic analyses taken to determine the possible sources of variation are outlined below.

### Read characteristics by library

First we assessed the general characteristics of the reads obtained for the different libraries. The results in Table 1 shows that the sequenced libraries differ considerably in the number of reads, depending on the sequencing technology, the particular version of the sequencing machine used, and whether or not a barcode or index had been used for parallel sequencing with other libraries. The most striking difference seen in terms of read number was between libraries from 454 sequencing, which have less than 100,000 reads, compared to the libraries from the two other technologies, which have millions of reads.

**Figure 1:**
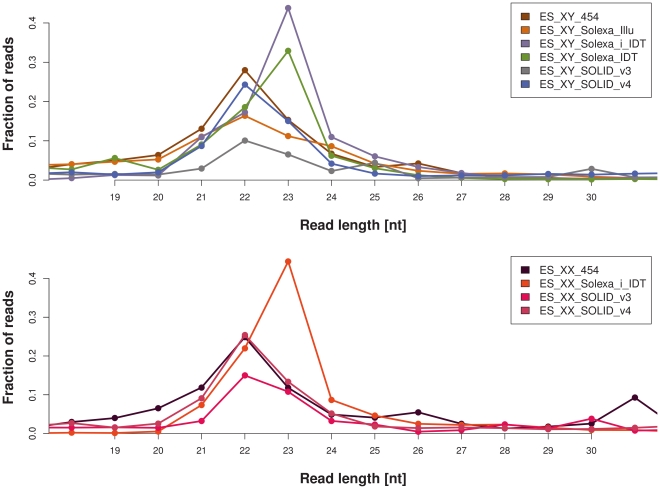
Read length distributions after adapter removal. The upper panel shows the E14 XY libraries while the lower panel displays the PGK XX libraries. See Table 1 for details about the libraries.

One of the specificities of small RNA libraries is that the reads generated by the sequencing machines are usually longer than the short RNAs that were sequenced. Thus, most sRNA-seq reads contain a part of the 3′ adapter sequence at their end, which must be removed prior to further processing of the data (see Materials and Methods for details). After adapter removal, the length distribution of the trimmed reads should correspond to the length distribution of the RNAs sequenced. Figure 1 shows the distribution of read lengths for each of the investigated libraries. Only the 19–30 nt size range is shown as this was the input for sequencing. The highest fraction of reads in each of the investigated libraries is 22 nt or 23 nt in size, as expected since this is the described size range of microRNAs, which likely make up the bulk part of sequenced small RNAs in ES cells [9]. Intriguingly, the most prominent read size, either 22 nt or 23 nt, differs between libraries. The three Solexa libraries sequenced with the IDT 3′ adapter (ES_XY_Solexa_i_IDT, ES_XY_Solexa_IDT, ES_XX_Solexa_i_IDT) have a peak of 23 nt reads, while for the other seven libraries, the peak clearly is at 22 nt. Furthermore, some libraries show a sharp and narrow peak at 22–23 nt, while other libraries, especially those generated by the SOLiD technology, show a larger spread of read lengths. Finally, certain libraries revealed unexpected, secondary peaks at certain read lengths. In summary, these data illustrate that the use of different adapters and protocols can lead to differences in the small-RNA sets sampled for sequencing.

### Alignment and annotation of reads

Next we compared differences between libraries with respect to reads that could be mapped to the mouse genome reference sequence (assembly mm9). The general category of each aligned read was investigated, based on the annotation of genomic features (genes, miRNAs, other non-coding RNAs [ncRNAs]) retrieved from the Mouse Genome Database (MGD), the Rfam database ([11], release 10.0), miRBase ([12], release 16) and the RepeatMasker track from the UCSC Genome Browser [13].

**Figure 2:**
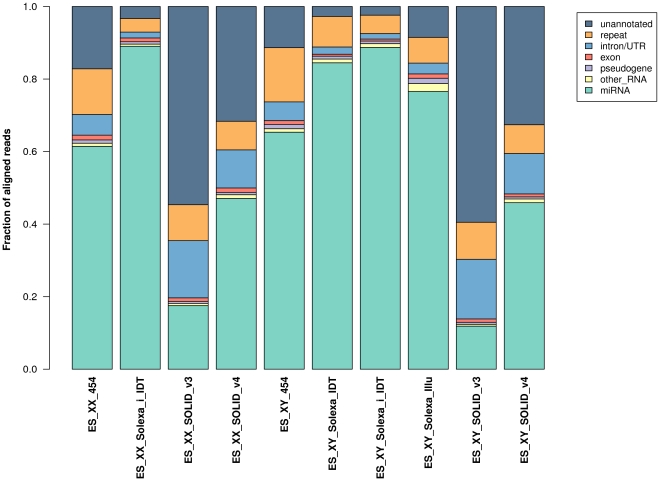
Categories of all aligned reads for the libraries investigated. The feature type of reads overlapping multiple genomic features was assigned by prioritising the feature types in the order: microRNA>other ncRNA>pseudogene>exon>gene>LINE>other repeat.

Figure 2 shows the fractions of aligned reads thus annotated for the most common feature categories (see Materials and Methods for details). In most libraries, the largest fraction of aligned reads overlaps with the positions of pre-miRNAs, as expected given that most of the small RNAs isolated from mouse ES cells should correspond to miRNAs. Several aligned reads do not overlap any type of genomic feature and are thus terms “unannotated”. The fraction of unannotated reads was especially high for two libraries sequenced using the SOLiD platform, where over 50% of the aligned reads could not be annotated. A likely explanation for this is that all reads generated from the SOLiD platform are reported directly upon sequencing, whereas the 454 and Solexa sequencing platforms include a pre-filtering step following sequencing. The extent of filtering could at least partly explain the differences observed in the fractions of unannotated reads.

**Figure 3:**
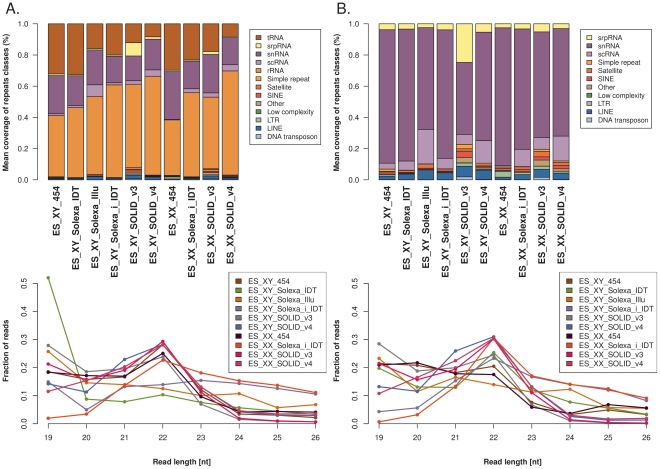
Annotation of repeats for the libraries investigated. **A.** Coverage of all repeats classes (in proportion). **B.** The three main repeats classes (RNA, rRNA, tRNA) were discarded to highlight the annotation for the other classes. **C.** Size distribution of reads aligned on all the repeats classes. **D.** Size distribution of the reads aligned on repeats classes excluding RNA, rRNA and tRNA.

We also assessed reads that mapped to multiple sites in the genome and found that the fraction of reads annotated for repetitive elements is similar among samples sequenced with the same technology, but shows some differences between technologies and protocols. Repeat profiling was performed with reads that could be aligned on the mm9 genome but were not annotated as pre-miRNAs. As for the miRNA profiling, only aligned reads in the 19–26 nt size range length were investigated. An aligned read was assigned to a repeat class if the read aligned position did not differ from the annotated position by more than 2 bp. Finally, the read counts for each repeat class were normalized by the number of genomic instances of the class in order to assess the mean coverage of each repeats class (Figure 3). The repeat profiles of all the samples are highly similar, with a high proportion of rRNA and tRNA sequences, and the length distribution of repeat-associated reads shows a peak at 22 nt. Repeat elements of the snRNA, scRNA and srpRNA classes on average show the highest coverage of small RNA reads, followed by the LINE class. Thus, although we found overall similarities in small RNA sequences derived from repetitive sequences between protocols, some important differences were also noted.

### Differences in miRNA expression

To gain a more precise view of the comparability of sRNA-seq data between libraries, we computed the number of counts per mature microRNA (miRNA) or microRNA star (miR*) from the aligned reads (see Materials and Methods for details). For the comparison, we considered each mature miRNA and miR* that had at least one read associated with it, in at least one of the 10 samples. The total set consists of 835 investigated miRNAs and miR*s. Figure S2 shows the number of libraries in which each miRNA (or miR*) had at least one read associated with it. In this way we could deduce that 32.1% of the miRNAs and miR*s were detected in all ten libraries investigated here. A large proportion of the reads in each library (median = 38.9%) corresponds to the miR-290 cluster on chromosome 7, which has previously been described to be highly expressed in undifferentiated ES cells [14]. Table S1 holds the exact read counts of each miRNA in the 10 libraries.

**Figure 4:**
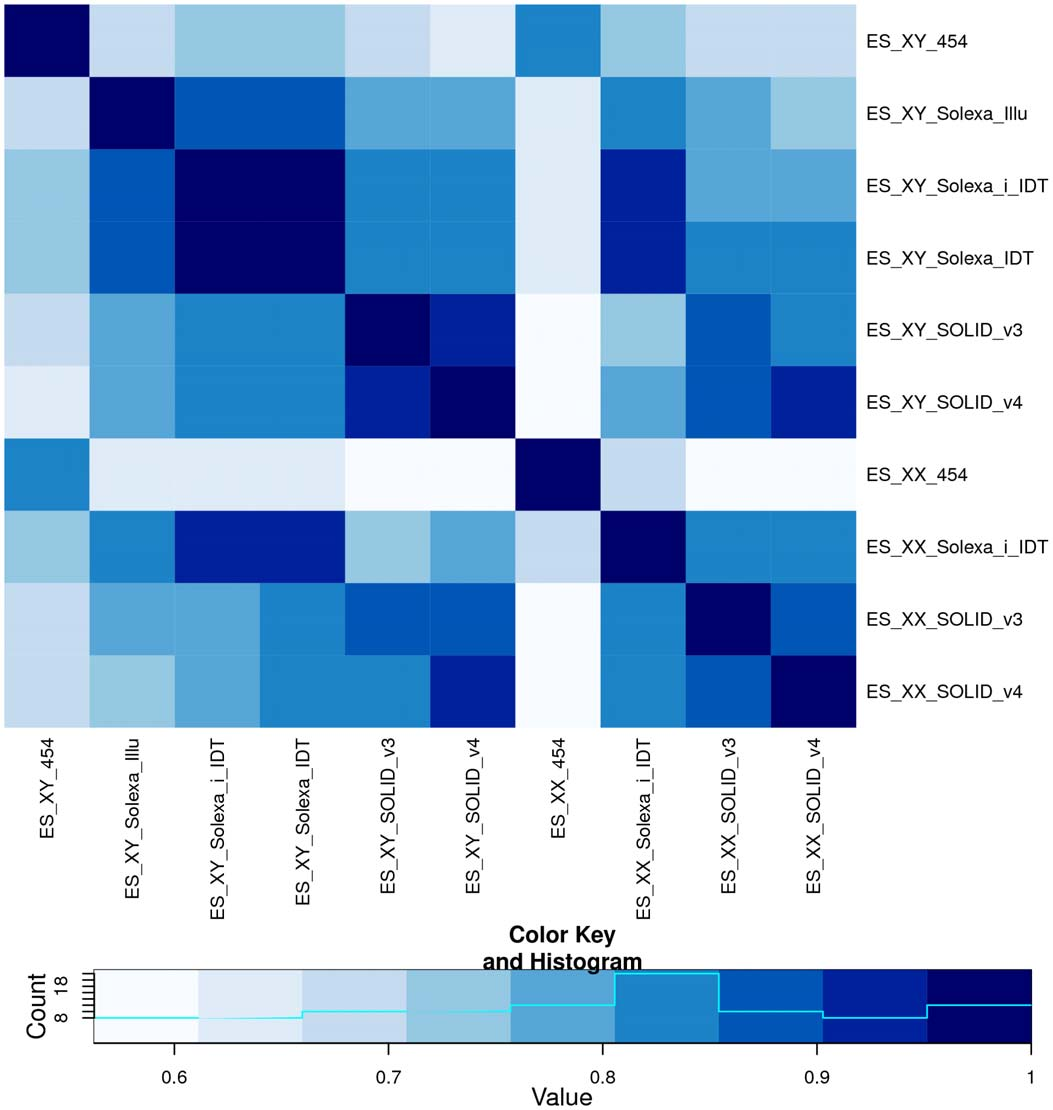
Correlation of miRNAs expression. Heatmap showing the pair-wise Spearman rank correlation between the miRNA read counts of the 10 libraries. The colour key at the bottom indicates which colour represents which correlation coefficient range.

In order to assess the general degree of similarity between the 10 libraries, we calculated the pair-wise Spearman (rank) correlation between the actual miRNA read counts per library (Figure 4). Overall miRNA read counts are highly correlated between the 10 libraries, with the Spearman correlation coefficient (CC) ranging from 0.563 to 0.982. The 454 libraries were highly correlated (CC = 0.811) to each other, but the correlation with any library generated by either of the other two sequencing technologies was low (CC<0.713). The reduced correlation between 454 libraries and SOLiD or Solexa, can partly be explained by the lower sequencing depth of 454 sequencing compared to Solexa or SOLiD sequencing. An almost perfect correlation (CC>0.936) was found among the sets of Solexa libraries that were sequenced on the same platform, using the same 3′ adapter. The four SOLiD libraries each showed a very good correlation with other SOLiD libraries (CC>0.846), but a much lower correlation to libraries generated by either 454 or Solexa sequencing technologies. The inter-correlation between Solexa libraries was even higher than that found between SOLiD libraries.

**Figure 5:**
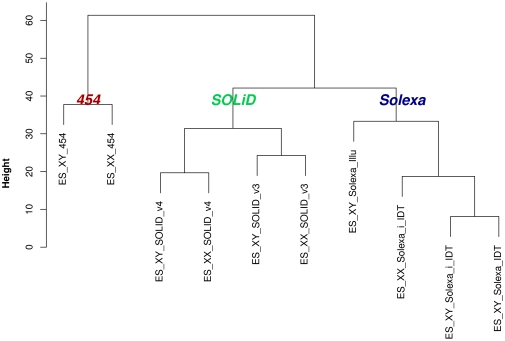
Clustering of sRNA-seq librairies. Hierarchical clustering dendrogram visualising the pair-wise distances between the 10 libraries after normalisation of miRNA read counts. The library identifiers correspond to the identifiers used in Table 1.

In order to compare miRNA expression levels and profiles between libraries, miRNA read counts per library were normalised using the two-step procedure described in Anders et al. [14]. Based on the normalised read counts, we performed a hierarchical clustering of the libraries to visualise the general proximity of the libraries to each other (Figure 5). In general, even though libraries were grouped by cell type for normalisation, the hierarchical clustering reveals that libraries in fact cluster by sequencing technology and protocol rather than by cell type. Firstly, the samples are grouped by the sequencing technology used, Solexa, SOLiD or 454. Except for the cluster containing the two 454 libraries, the clusters show further sub-divisions. The Solexa libraries group together based the choice of 3′ adapter. The cluster containing the SOLiD libraries also sub-divides into two groups, based on the library preparation protocol and the version of the sequencing machine (version 3+/SREK kit versus version 4/STaR-Seq kit).

**Figure 6:**
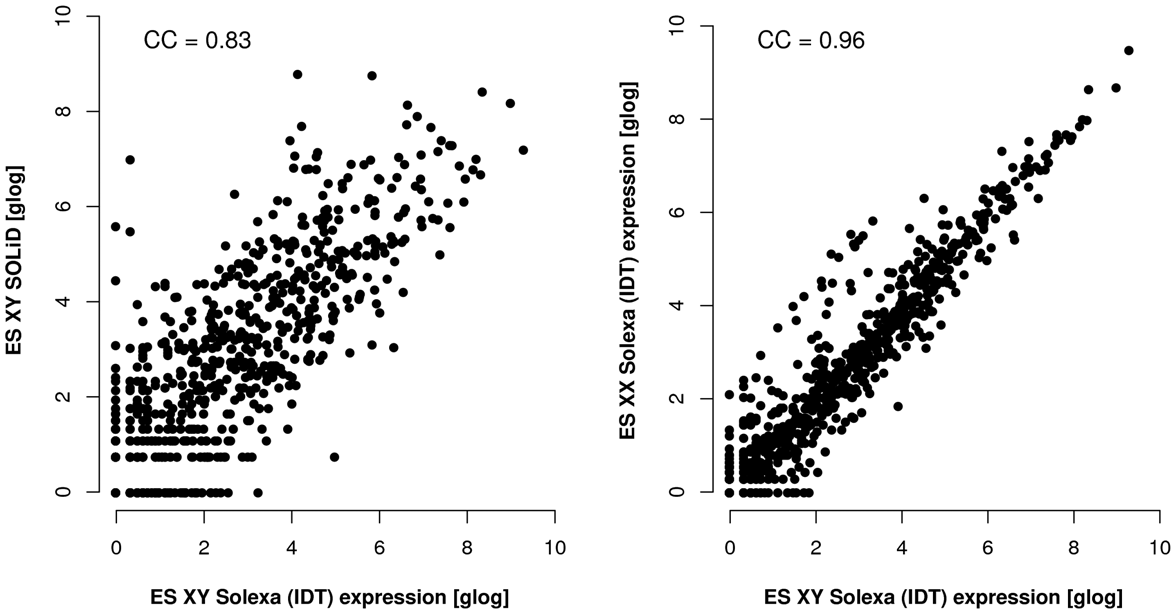
Comparison of miRNA expression levels. Scatter plots comparing the normalised miRNA expression levels (on a generalised logarithmic scale) between pairs of libraries. The libraries are named as in Table 1. A. Libraries were generated from the same cell line (E14 ES XY) but using different sequencing platforms (Solexa vs. SOLiDv4). B. Libraries were generated from different cell lines (XY vs. XX), but both using the Solexa sequencing platform. CC: Spearman correlation coefficient.

Figure 6 shows scatter plots comparing the miRNA expression levels between pairs of libraries after normalisation. The SOLiD and Solexa libraries in the left panel of Figure 6 (ES_XY_Solexa_IDT and ES_XY_SOLiD_v4 both made from the same cell line, E14 male ES) have a comparable sequencing depth, but nevertheless it can be seen that several miRNAs are only observed in one or the other of the two libraries. This finding indicates that the detection of certain miRNAs from the same cell line, largely depends on the sequencing platform used and in particular on the library preparation protocol (Figure S4).

Given the observed differences, we next investigated the potential influence of the library preparation protocol by comparing libraries generated using the same sequencing technology. Figure S3 shows scatter plots comparing the miRNA expression levels between pairs of libraries from the same cell line generated using the same sequencing technology (Solexa for left panel and SOLiD for right panel), but using different library preparation protocols or different versions of the technology (see Table 1 for details). The correlation is higher (respectively 0.98 for Solexa and 0.91 for SOLiD) than that seen in Figure 6. However, there are still miRNAs specifically expressed in only one of the two libraries. Furthermore, the use of an “index” for multiplexing (library ES_XY_Solexa_i_IDT) did not seem to affect the distribution of miRNA read counts.

Finally, it can be seen that two libraries made from different biological samples, male versus female ES cell lines, but generated with the same library-preparation protocol and sequencing platform (Solexa with IDT 3′ adapter, right panel of Figure 6) show a higher correlation than two libraries from the same sample but sequenced on different platforms. A higher correlation is found between the values from these different ES cell lines compared to libraries generated from the same biological sample but different library preparation and sequencing technology. Taken together, these data demonstrate unequivocally that the technological variability introduced by different sequencing platform and library-preparation protocol outweighs the biological variability between male and female ES cell lines.

Although the overwhelming differences between libraries were due to library preparation and sequencing technology, we were also able to detect some differences between male and female ES cell samples (Figure S5). Using all of the samples listed in Table 1, 25 miRNAs and miR*s were found to be differentially expressed between male and female ES cells (corrected *p*-value *p*<0.05), with 6 of these being located on the X chromosome (Table S2). Thus, despite the aforementioned technical source of variation, there also is a biologically meaningful variation between the libraries investigated.

In agreement with previous findings [5], we find that observations based on fold-changes may be more transferable across sequencing platforms than actual read counts. Figure S6 shows the fold-changes between miRNA read counts in female and male ES cells (CC = 0.87, compared with CC = 0.84 in the left panel of Figure S4).

### miR/miR* ratios can be influenced by the sequencing protocol

The percentage of mature miRs is usually much higher than that of the miR*s in small RNA populations, presumably because the passenger strand miR*s are rapidly degraded [15]. We investigated the detectability of these low-level miR* entities, together with their miR counterparts, in the different libraries generated here by visualising the percentage of reads for each miR and miR* using the UCSC genome browser. An example of this can be found in Figure S7, where the expression of the mmu-miR-290 cluster, located on chromosome 7, in male ES cells generated by different technologies is shown. The Figure S7 illustrates some important differences between the libraries. In two of the libraries, mmu-miR-295 is the most highly expressed miRNA, while in other libraries mmu-miR-294 or mmu-miR-293 are expressed more highly than mmu-miR-295. Importantly, in two of the libraries examined, namely ES_XY_Solexa_IDT and ES_XY_Solexa_i_IDT, the star form, mmu-miR-293*, appears to be more highly expressed than the corresponding mature miRNA. This highly unusual pattern is not consistent with previous findings on the biogenesis of miRNAs in ES cells [16], or with findings in other libraries. The common feature of these libraries showing unexpectedly high proportions of miR* entities is the use of an “IDT” 3′ adapter [7]. The Solexa libraries prepared with the standard Illumina 3′ adapter, as well as the libraries made using the other two sequencing platforms do not show such abnormally high level of miR*s, implying that the IDT 3′ adapter sequence could be at least be partially responsible for this enrichment of miR* sequences.

To investigate this further, we examined the ratio of miR/miR* forms in all of the libraries. Table S3 presents the numbers, per library, of several miR/miR* pairs for which miR* reads were higher than mature miRNA reads. In the 454 libraries, unexpected ratios were only seen for three miR/miR* pairs. However, the 454 libraries are of considerably smaller size than the Solexa or SOLiD libraries, for which between 18 and 24 miR/miR* pairs were affected.

Most miR/miR* pairs that showed unexpected star/mature ratios in any library, were observed in a specific group of libraries, such as the unusual mmu-miR-293 pattern in the IDT-libraries described above (Figure S7). However, in the case of a few miRNAs, namely mmu-miR-140, mmu-miR-154, and mmu-miR-28, considerably more reads were found for the miR* form than for the mature miR in most of the libraries investigated (except for the 454 libraries). However, in these cases, our analysis indicates that the mature forms of these miRNAs may have been mis-annotated in the miRBase database (release 16). The miR/miR* nomenclature provided by miRBase (until release no. 16) was based on the abundance of the mature product. However, recent publications investigating the potential functional role of the miR* species proposed that the relative abundance of the dominant mature form and the miR* form may depend on tissue, stage and species [17]. Accordingly, the miR/miR* nomenclature was withdrawn from the latest release (no. 17) of miRBase, in favour of the −5p/−3p nomenclature.

Finally, in the case of three other miR/miR* pairs, namely mmu-miR-299, mmu-miR-872, and mmu-miR-877, unexpected miR/miR* ratios were seen consistently in libraries generated by the SOLiD platform, but not with the other technologies. No miR/miR* pairs were found to be specifically affected in the Solexa or the 454 libraries. The decoded sequences of most SOLiD reads counted for miR-299* or mir-877* were predicted to have terminal secondary structures by the mfold web server [18] with default parameters. The finding that SOLiD sequencing shows a positive bias for reads with 3′-terminal secondary structures agrees with previous observations [5].

In summary, these findings indicate that the miR*/miR ratios can be influenced by several variables, including the choice of adapter, as well as the library preparation. The precise basis for these differences is unclear. Furthermore, it is becoming clear that for some miRs the level of miR* entity can be quite high in ES cells whatever the protocol or technology used, and this likely has a biological basis.

### Profiling of reads derivative from repetitive elements

We also investigated putative differences between sequencing protocols and technologies at the level of small RNAs generated from repetitive elements in ES cells. As mentioned previously, repeat element content seemed to show some differences between ES cell libraries, which could be due in part to sequencing technologies or protocols. To investigate this further, we looked in detail at the manner in which Long interspersed elements 1 (LINE-1 or L1) derived small RNAs varied between our libraries.

Recent studies involving deep sequencing of small RNA libraries from mouse ES cells or oocytes have provided evidence that small RNAs can be derived from certain families of highly repetitive sequences [19], [20], [21]. The mechanisms by which such repeat-derived small RNAs are generated and their role if any, remains obscure. In the case of LINE-1 elements it has been proposed that these repeat-derived small RNAs may reflect a global RNAi-type mechanism involved in regulating mRNA expression and/or L1 activity [22]. Our previous work characterised the L1 profiles generated using 454 sequencing [10], [22]. Here, we investigated the L1 repeat-derived small RNAs profiles in our ten samples.

**Figure 7:**
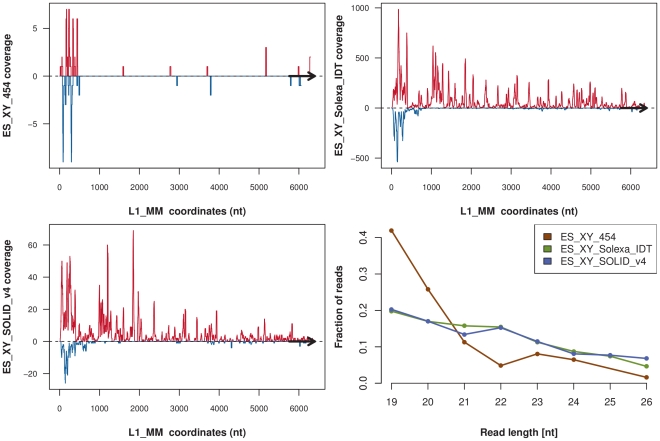
Reads coverage of L1 consensus sequence. The reads with a size of 19–26 nt were aligned on the L1_MM consensus sequence extracted from Repbase. The coverage from the ES_XY_Solexa_IDT, ES_XY_454 and ES_XY_SOLiD_v4 libraries, on the sense orientation is represented in red, whereas the coverage in antisense orientation is represented in blue. The size distribution of the reads aligned on the L1_MM consensus is shown for the three libraries.

In order to identify sequences that match different families of L1 elements, the reads were aligned to the consensus sequences of Repbase [23] (see Materials and Methods for details). The read coverage of the L1 consensus for the ES_XY_Solexa-i_IDT, ES_XY_454 and ES_XY_SOLID_v4 samples is shown in Figure 7. The three samples from the three technologies present a similar profile. We observed that many of these L1-derived small RNAs mapped to the promoter region of the consensus sequence, in both the sense and antisense orientations, whereas the ORFs of the L1 consensus sequence are mainly covered by reads aligned in the sense orientation. These reads are detected in all 10 libraries. The read size distribution differs between libraries however. The 454 library contains twice as many reads that are 19 nt long as the SOLiD or Solexa libraries, and a lower proportion of reads in the 22 nt size range. On the other hand the majority of reads in the Solexa and SOLiD libraries are 19–22 nt long. The basis for these differences in L1 sRNA read length between the 454 and Solexa libraries is unclear. One explanation might be that they are degradation products, in the homemade protocol used for the 454 library generation.

## Discussion

In this study, we have assessed similarities and differences in small RNA sequencing profiles that are based on library preparation, as well as biological differences. To this end, we have examined small RNA populations obtained from mouse ES cell samples and analysed using different NGS technologies. In general, we find that sRNA-seq libraries generated from the same biological sample showed a reasonably good correlation, even when different protocols were used for their sequencing. However, we also find that the library-preparation protocol (and accordingly the sequencing technology used) could have a profound impact on the miRNA expression profiles observed using NGS techniques. The impact of the sequencing technology on its own was not assessed in this study, as no samples from exactly the same library preparation were sequenced on the different platforms. Such type of comparisons, however, have been performed previously [4]. Linsen et al. compared micoRNA expression profiles from rat brains across three different libraries preparations, poly(A) tailing, *modban* adaptor (IDT) ligation [7] and Small RNA Expression kit (SREK-ABI first version), and three sequencing platforms, Roche 454, ABI-SOLiD and traditional capillary dideoxy sequencing. They analysed the 10 most frequently sequenced microRNAs of each library-preparation method and concluded that biases observed are largely independent of the sequencing platforms but strongly determined by the method used for small RNA library preparation. Here, we extend upon these results adding more recent protocols, platforms (Roche-454, Illumina-Solexa and ABI-SOLiD) and small RNA types. We also show that the library-preparation protocol used has serious implications in the interpretation of data, particularly when biological differences between small RNA populations and pathways are being sought. In contrast, the use of an “index” for multiplexing had negligible effects on the profiles obtained and seems to be really better that barcodes (Figure S3).

In summary, libraries from different cell lines that were sequenced on the same type of platform/machine type were found to be far more similar to each other, than to libraries from the same cell line (identical biological sample) sequenced using different technologies. Both of the ES cell lines investigated in this study corresponded to undifferentiated embryonic stem cells, and *a priori* they were not expected to show striking differences, even though one is female and the other male. Indeed, when considering data from all of the libraries examined here we found that just 25 microRNAs were differentially expressed between the two cell lines, with one quarter of these originating from the X-chromosome. This demonstrates that biologically relevant differences could be found between male and female ES cells, but these differences were minor compared to some of the differences found for the same cell line sequenced with different technologies.

Furthermore we also found that even when using the same NGS technology, different library preparation protocols could lead to apparent differences in miRNA expression levels and, in certain cases striking differences in the detection of miR*s. These differences were clearly technical in some cases, although the molecular basis of this remains unclear. In other cases, unusual proportions of miR* entities were found in all of the libraries investigated, suggesting that this may be a biologically relevant result and a mis-annotation of miRbase.

The profiles of small RNAs from repetitive elements generated from the same biological samples showed a good correlation between the three sequencing technologies. The main difference observed concerned the proportion of tRNAs and rRNAs. The mean coverage of ncRNA or LINE repetitive regions is comparable between libraries. However, only the reads aligned less than 5 000 times were used for this analysis of repeat elements. This filtering may have influenced some of the results, such as the proportion of reads aligned on simple repeat elements, according to the number of reads from each library. Nevertheless, all the library preparations and sequencing approaches led to similar coverage profiles over the L1_MM consensus sequence. This consistency between sequencing technologies, which contrast with the differences we found for miRNAs, may be due the fact that here we are looking at whole small RNA populations, whereas in the case of miRNAs we are looking at discrete small RNAs characterized by their size, genomic region and their orientation.

In conclusion, the comparative analysis we report here suggests that caution needs to be applied to the interpretation of small RNA sequencing data generated using different technologies or protocols, particularly in terms of miRNA expression levels, as clearly the conclusions depend on the technology used in each case. Thus far, no single protocol and sequencing technology has been shown to best represent biological reality. Probably every type of library preparation and sequencing technology introduces a certain amount of bias and samples a slightly different pool of small RNAs in a cell. Thus, when using second-generation sequencing for comparing small-RNA populations between different biological samples, it may be advisable to consistently use the same sequencing technology and library preparation protocol (adaptors and indexes) for all libraries to be investigated. The libraries will thus all be affected by the same bias, which therefore will have minimal influence on the comparison of results from different biological samples. Conversely, it is important to consider that diversifying sequencing technologies and protocols may be helpful for generating complete inventories of small RNAs in any given sample.

Finally, it should be noted that certain protocols utilised for preparing sRNA-Seq libraries are also used for generating libraries from messenger RNAs (mRNAs). Hence, similar biases from adaptors and barcoding may also affect the results of RNA-Seq in general and need to be taken into account in the experimental design of sequencing studies.

## Materials and Methods

### Cell lines

Female PGK and male E14 Embryonic Stem (ES) cell lines (from Dr E. Heard laboratory) [10] were cultured in Dulbecco's Modified Eagle Media (DMEM) (Invitrogen), containing 15% FCS (Bio West), 1000 U/ml LIF (Chemicon), 0.1 mM beta-mercaptoethanol (Invitrogen), 0.05 mg/ml of streptomycin (Invitrogen) and 50 U/ml of penicillin (Invitrogen) on a gelatin-coated support in the absence of feeder cells. The culture medium was changed daily. All cells were grown at 37°C in 8% CO2.

### Sequencing

Total cellular RNA samples (5–10 µg), prepared using Trizol reagent (MRC Molecular Research Center), were processed into sequencing libraries using: 1.) a homemade protocol [9] for the 454 technology and sequenced at Genoscope (Evry, France), 2.) adapted Illumina protocols for Solexa technology and sequenced by Fasteris ((http://www.fasteris.com, Switzerland), and 3.) the Small RNA Expression Kit (SREK, Life Technology, version C) and the SOLiD Total RNA kit (STaR-Seq, Life Technology) for the SOLiD technology and sequenced at Institut Curie (Paris, France) or Life Technology (USA). The raw and processed data are publicly available at GEO, series record GSE35368 (http://www.ncbi.nlm.nih.gov/geo/).

### Adapter removal and read alignment

The length of reads output by the sequencing generally exceeded the length of the investigated RNAs. Thus, remaining parts of the adapter sequence had to be removed by identifying overlaps between the end of the reads and the beginning of the provided adapter sequence. This task was done using different tools specific to each sequencing technology: a script from M. Zavolan (University of Basel, Switzerland) for the 454 data, custom scripts from Fasteris (Switzerland) for the Solexa data, a custom script from N. Socci (Memorial Sloan-Kettering Cancer Center) for the SOLiD data. Each time, we made sure to find the maximal overlap between beginning of the 3′-adapter and end of read sequence and to cut exactly in front of the first adapter base. The trimmed sequencing reads of each library were then mapped to the mouse genome reference sequence (assembly mm9) and to the RepBase (v16.03) consensus sequences using the alignment tool Bowtie ([24], v0.12.7). Respectively, two mismatches in nucleotide space or colour space were allowed for the mapping of the 454 and the SOLiD data, while for Solexa the sum of qualities of mismatching bases was required to not exceed 50. Only the best alignments are reported for each reads. The reads with up to 5 000 repeated alignments on the genome were used for the repeats analysis. The reads aligned on different repeats classes were not discarded from the analysis.

### Data analysis

The read alignments were rigorously checked for quality. For each library, we computed the counts per mature miRNA and miR* from the read alignments. The genomic positions of mature miRNAs and miR* were obtained from the database miRBase ([12], release 16). Aligned reads of length 19–26 nt were considered to correspond to a mature miRNA (or miR*) only if 1.) the aligned position did not differ from the annotated position of the mature miRNA (or miR*) by more than 2 bp and 2.) the reads had at most as many genomic match positions as the number of genomic copies of the respective mature miRNA (or miR*). Each aligned read was assigned to a genomic feature type if its aligned position overlapped the position of the annotated feature by 70% or more. The feature type of reads overlapping multiple genomic features was assigned by prioritising the feature types in the order: microRNA>other ncRNA>pseudogene>exon>gene>LINE>other repeat.

In order to be able to compare miRNA expression levels and profiles between libraries, miRNA read counts per library were normalised using the two-step procedure described by Anders et al. [14]. Briefly, this normalisation consists of a division of the read-counts by estimated library-size factors followed by a variance-stabilising transformation. For the normalisation, libraries were pooled by cell type to estimate the within-group variation. Then, the normalized miRNA read counts were tested for differential analysis using the R package *DESeq* [14].

The hierarchical clustering applied is based on the Euclidean distance between pairs of libraries, and clusters are agglomerated using the complete-linkage method.

Repetitive element profiling was performed after removal of reads aligned to pre-miRNA regions. The remaining reads were annotated using the RepeatMasker track from the UCSC Genome Browser [13]. All read alignments were used to compute the mean coverage of the different repeats classes.

Finally, in order to identify sequences that match different families of L1 elements, the reads were aligned on the consensus sequences of repetitive elements extracted from Repbase (v16.03). The L1_Mm consensus sequence was used as the reference of mouse L1 repetitive sequence.

For analysing the sequencing data, we mainly used the BEDTools suite [25] to annotate the reads files, and the R statistical environment with packages from the Bioconductor project [26], in particular *girafe* [27], *Rsamtools* and *DESeq* [14].

## Supporting Information

Figure S1 **Schematic representation of small RNA cDNA library preparation adapted from Pfeffer et al.** [**7**] **.** The insertion of a barcode or index is specifically highlighted.
(TIFF)

Figure S2 **miRNA detection across the different libraries.** Histogram showing the number of libraries in which each miRNA (or miR*) is represented by one or more reads.
(TIFF)

Figure S3 **Comparison of miRNA expression levels between libraries from the same cell line and sequencing technology.** Scatter plots comparing the normalised miRNA expression levels (on a generalised logarithmic scale) between pairs of libraries generated using the same sequencing technology but different library preparation protocols or versions of the technology. The libraries are named as in Table 1. CC: Spearman correlation coefficient.
(TIFF)

Figure S4 **Comparison of miRNA expression levels between libraries from the same cell line and different sequencing technology.** Scatter plots comparing the normalised miRNA expression levels (on a generalised logarithmic scale) between pairs of libraries generated from the E14 cell line but using different sequencing technologies. Libraries are named as in Table 1. CC: Spearman correlation coefficient.
(TIFF)

Figure S5 **Comparison of miRNA expression levels between libraries from the two cell lines and the same sequencing technology.** Scatter plots comparing the normalised miRNA expression levels (on a generalised logarithmic scale) between E14 XY (y-axis) and PGK XX (x-axis) libraries generated using the same sequencing technology and library preparation protocols. The left panel contains a comparison of the two 454 libraries, the right panel displays the miRNA levels in two SOLiD libraries. The libraries are named as in Table 1. CC: Spearman correlation coefficient.
(TIFF)

Figure S6 **Fold-changes of microRNA reads counts between female and male ES cells across sequencing platform.** For all microRNAs and miR* investigated, we computed the fold-changes between female and male cells within one sequencing platform first (for Solexa: ES_XX_Solexa_i_IDT/ES_XY_Solexa_i_IDT; for SOLiD: ES_XX_SOLID_v4/ES_XY_SOLID_v4). The logarithms of the fold-changes determined for SOLiD are plotted versus those computed for Solexa. Top left, the correlation coefficient between the fold-changes is specified.
(TIFF)

Figure S7 **UCSC genome browser view of miR-290 cluster.** UCSC genome browser screenshot showing the expression of the miR-290 cluster on chromosome 7 in the 6 XY libraries (ordered as in Table 1). In each library, the percentage for each miRNA and miR* among all miRNA-associated reads is shown. Below, the genomic positions of the pre-miRNAs, as annotated in miRBase, are shown in red.
(TIFF)

Table S1 **microRNA reads counts.** Aligned reads of length 19–26 nt were considered to correspond to a mature miRNA (or miR*) only if the aligned position did not differ from the annotated position of the mature miRNA (or miR*) by more than 2 bp and if the reads had at most as many genomic match positions as the number of genomic copies of the respective mature miRNA (or miR*).
(XLS)

Table S2 **Differentially expressed microRNAs between ES male and female cells.** miRNA read counts per library were normalised and tested for differential analysis using the *DESeq* package [14].
(XLS)

Table S3 miR/miR* ratios. Number of miR/miR* pairs with more reads for the passenger star form than for the mature miRNA per library.
(XLS)
