# Supplementary figures and images for: Deep-Sequencing Protocols Influence the Results Obtained in Small-RNA Sequencing
Source: PLoS One. 2012 Feb 27;7(2):e32724. doi: 10.1371/journal.pone.0032724 (PMC3287988; doi:10.1371/journal.pone.0032724)

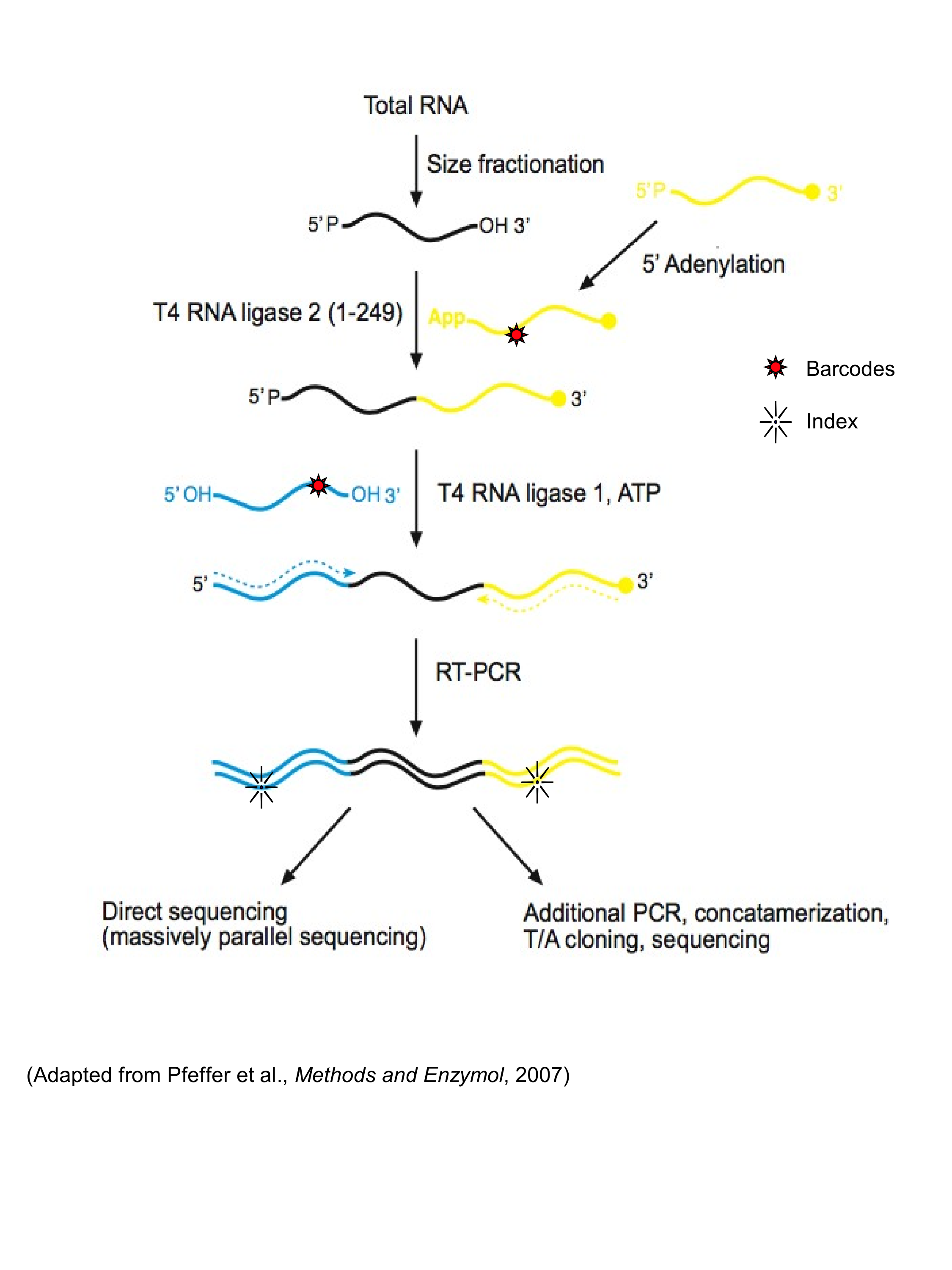

Supplement: Figure S1 — Schematic representation of small RNA cDNA library preparation adapted from Pfeffer et al. [7] . The insertion of a barcode or index is specifically highlighted. (TIFF) [file pone.0032724.s001.tif]

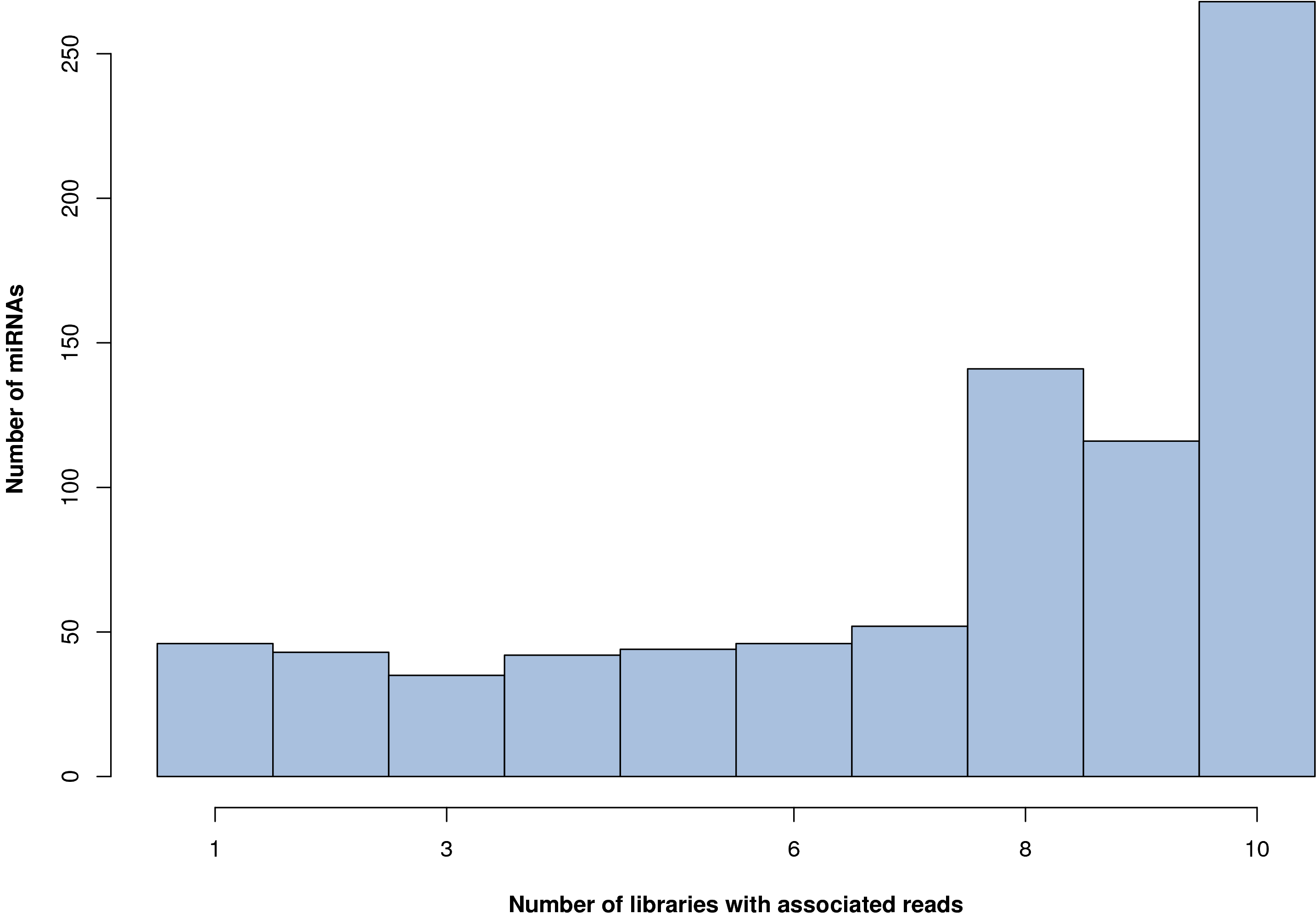

Supplement: Figure S2 — miRNA detection across the different libraries. Histogram showing the number of libraries in which each miRNA (or miR*) is represented by one or more reads. (TIFF) [file pone.0032724.s002.tif]

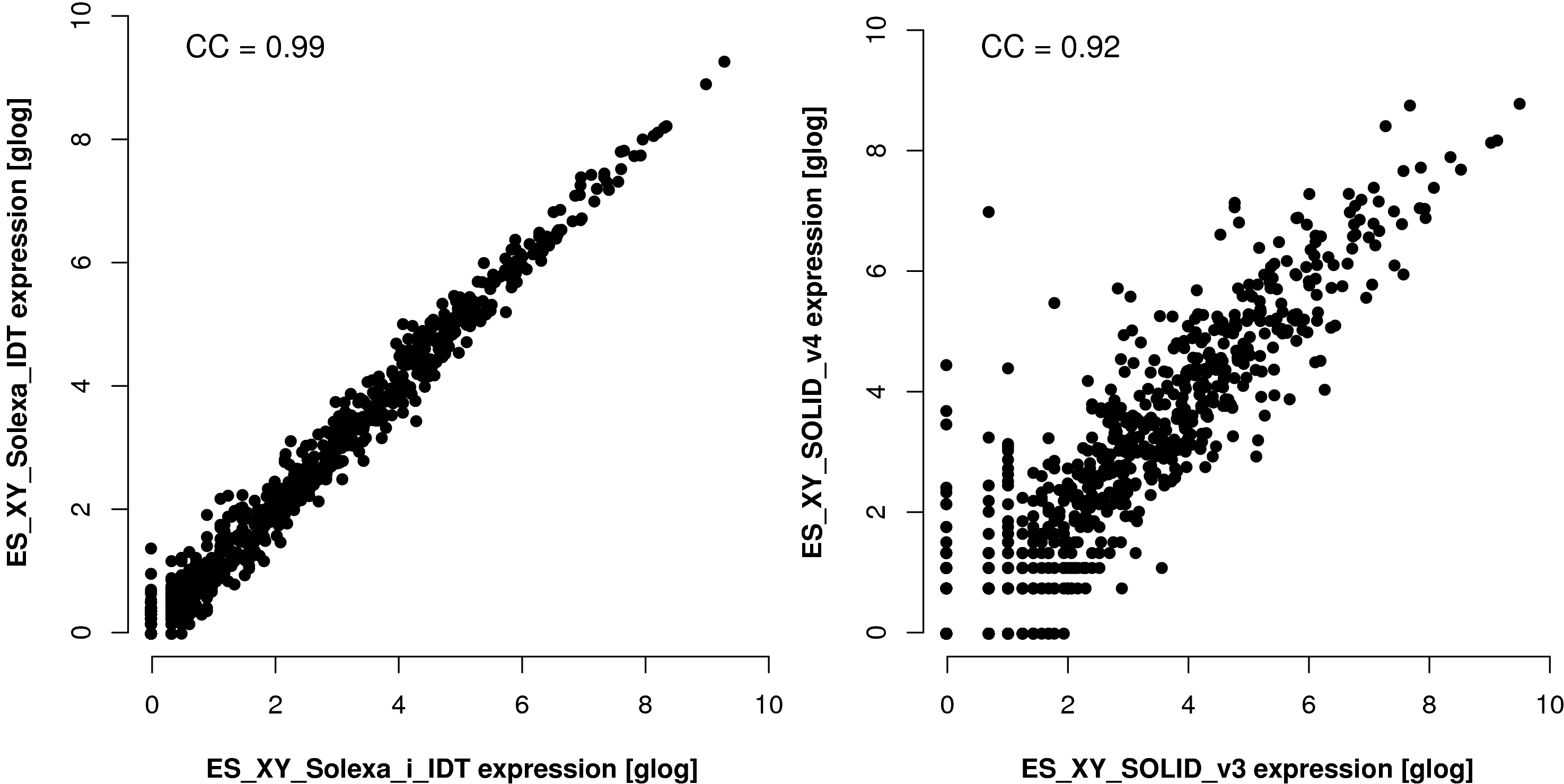

Supplement: Figure S3 — Comparison of miRNA expression levels between libraries from the same cell line and sequencing technology. Scatter plots comparing the normalised miRNA expression levels (on a generalised logarithmic scale) between pairs of libraries generated using the same sequencing technology but different library preparation protocols or versions of the technology. The libraries are named as in Table 1. CC: Spearman correlation coefficient. (TIFF) [file pone.0032724.s003.tif]

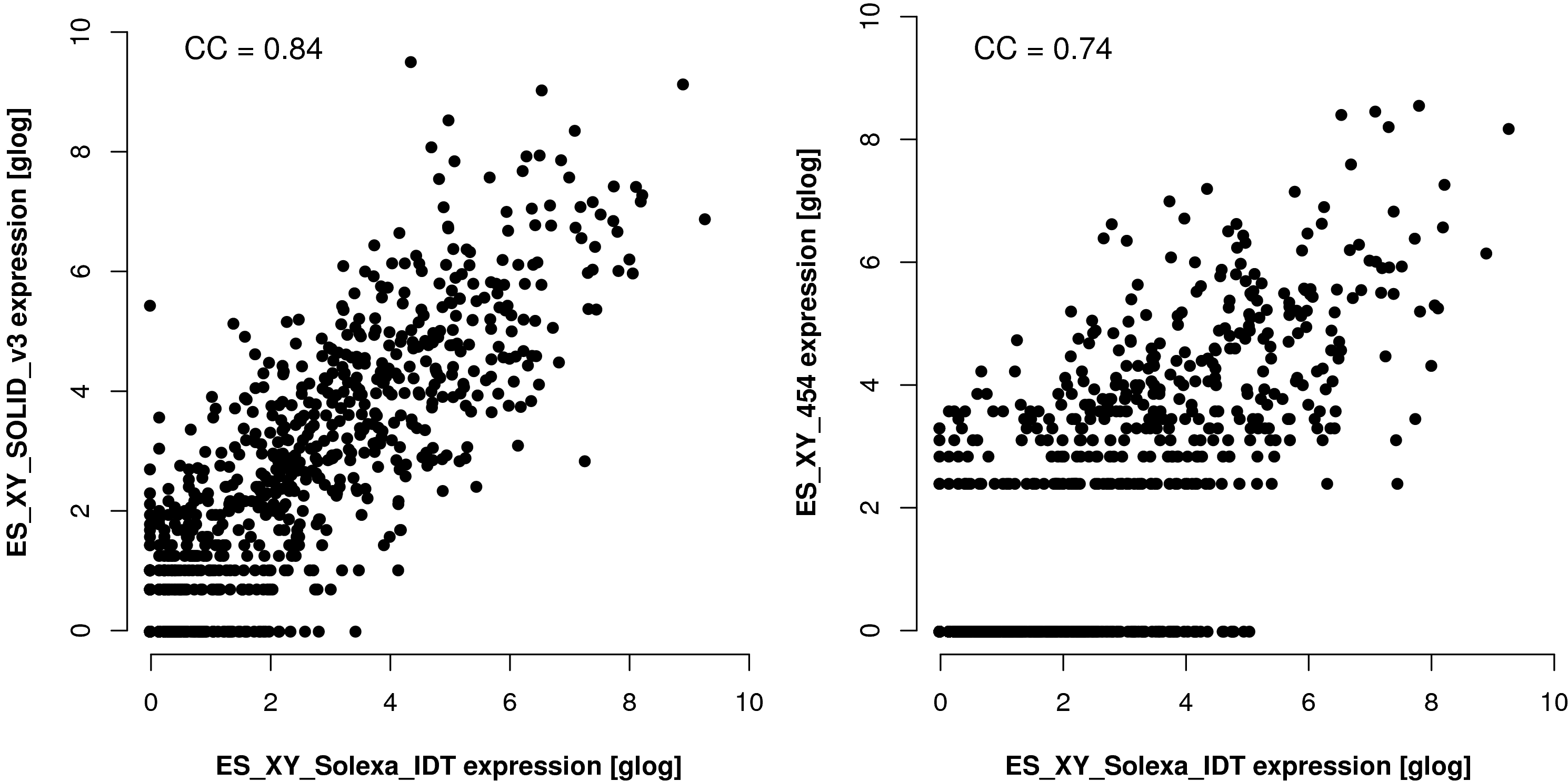

Supplement: Figure S4 — Comparison of miRNA expression levels between libraries from the same cell line and different sequencing technology. Scatter plots comparing the normalised miRNA expression levels (on a generalised logarithmic scale) between pairs of libraries generated from the E14 cell line but using different sequencing technologies. Libraries are named as in Table 1. CC: Spearman correlation coefficient. (TIFF) [file pone.0032724.s004.tif]

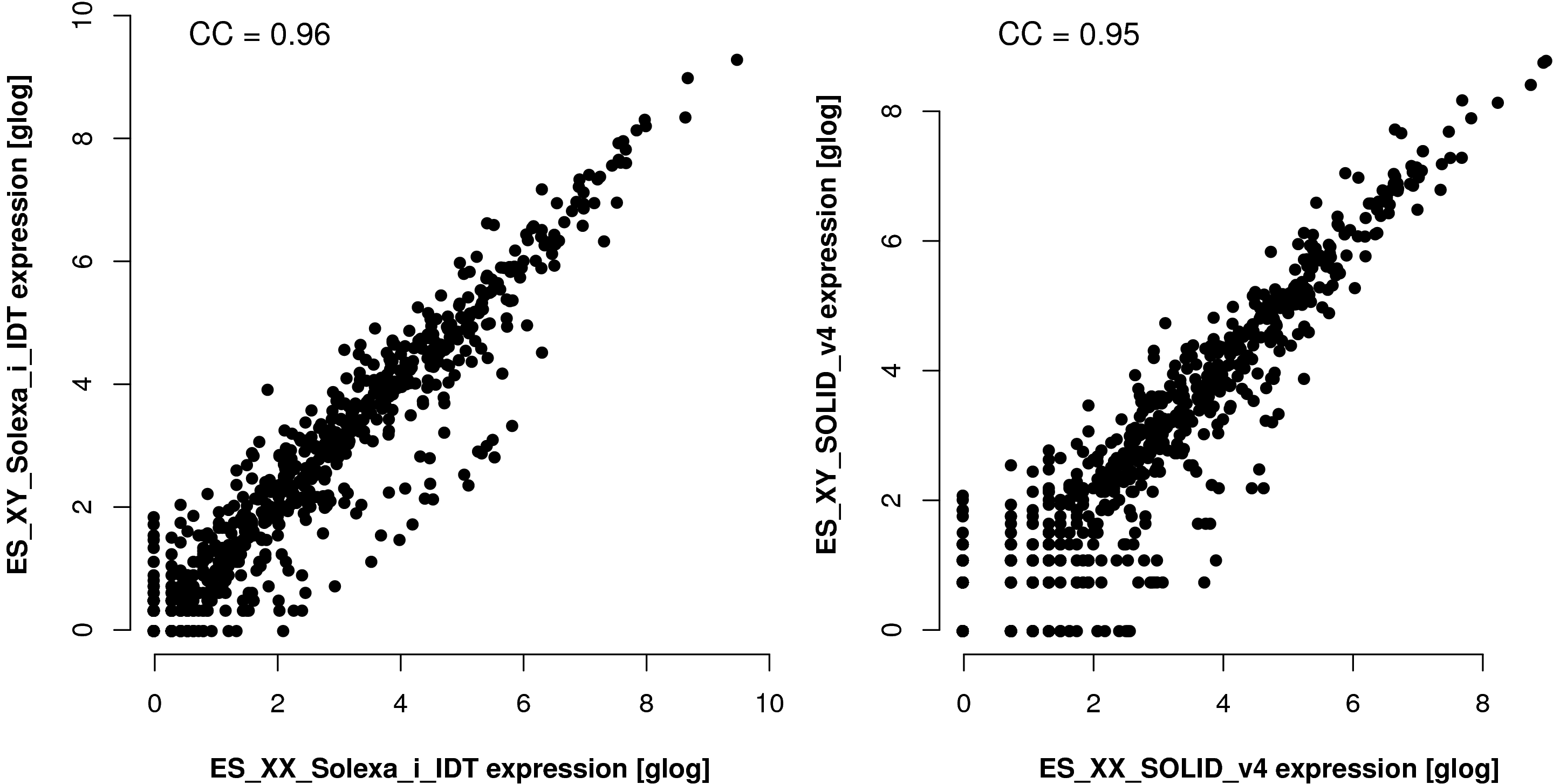

Supplement: Figure S5 — Comparison of miRNA expression levels between libraries from the two cell lines and the same sequencing technology. Scatter plots comparing the normalised miRNA expression levels (on a generalised logarithmic scale) between E14 XY (y-axis) and PGK XX (x-axis) libraries generated using the same sequencing technology and library preparation protocols. The left panel contains a comparison of the two 454 libraries, the right panel displays the miRNA levels in two SOLiD libraries. The libraries are named as in Table 1. CC: Spearman correlation coefficient. (TIFF) [file pone.0032724.s005.tif]

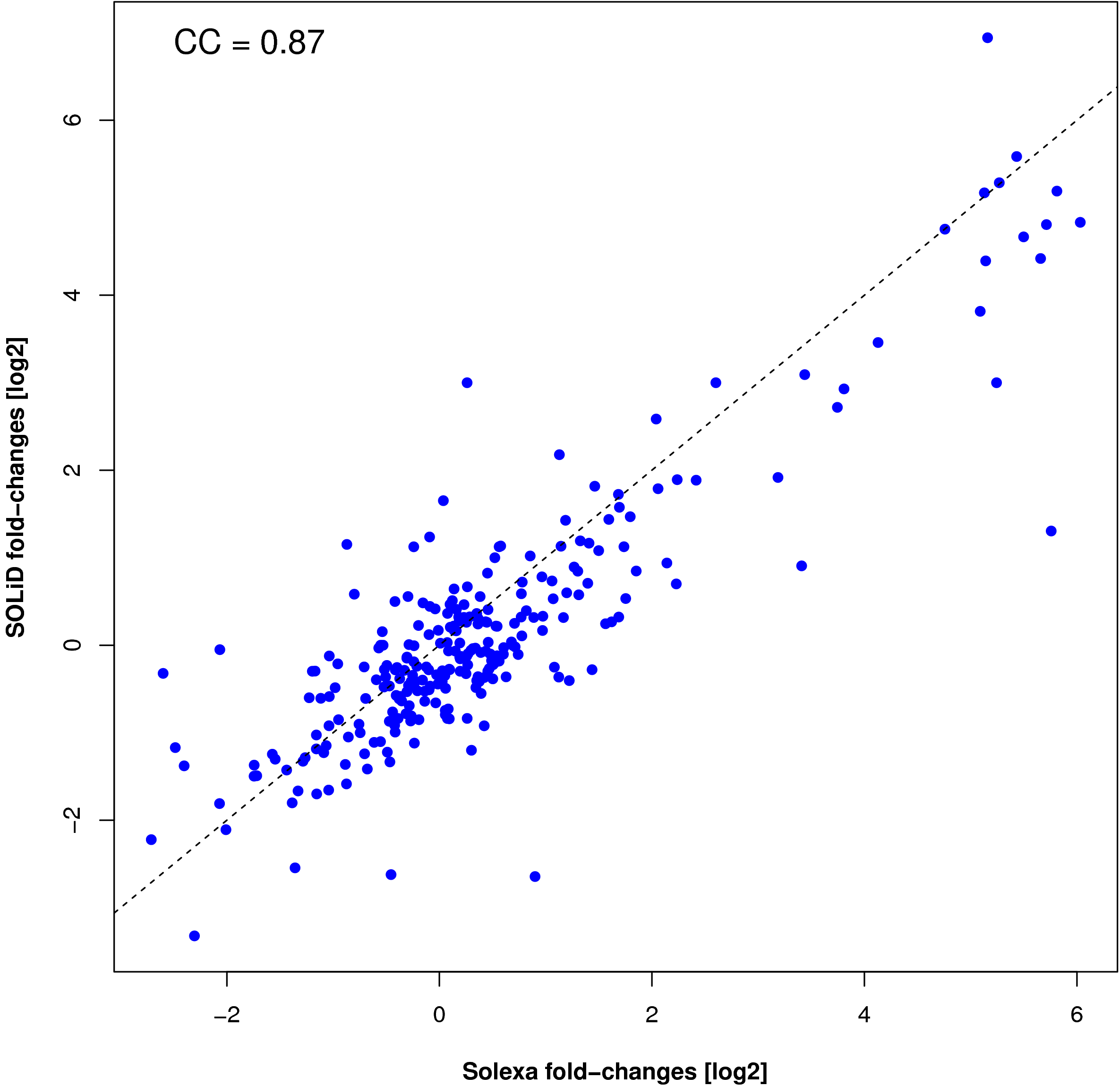

Supplement: Figure S6 — Fold-changes of microRNA reads counts between female and male ES cells across sequencing platform. For all microRNAs and miR* investigated, we computed the fold-changes between female and male cells within one sequencing platform first (for Solexa: ES_XX_Solexa_i_IDT/ES_XY_Solexa_i_IDT; for SOLiD: ES_XX_SOLID_v4/ES_XY_SOLID_v4). The logarithms of the fold-changes determined for SOLiD are plotted versus those computed for Solexa. Top left, the correlation coefficient between the fold-changes is specified. (TIFF) [file pone.0032724.s006.tif]

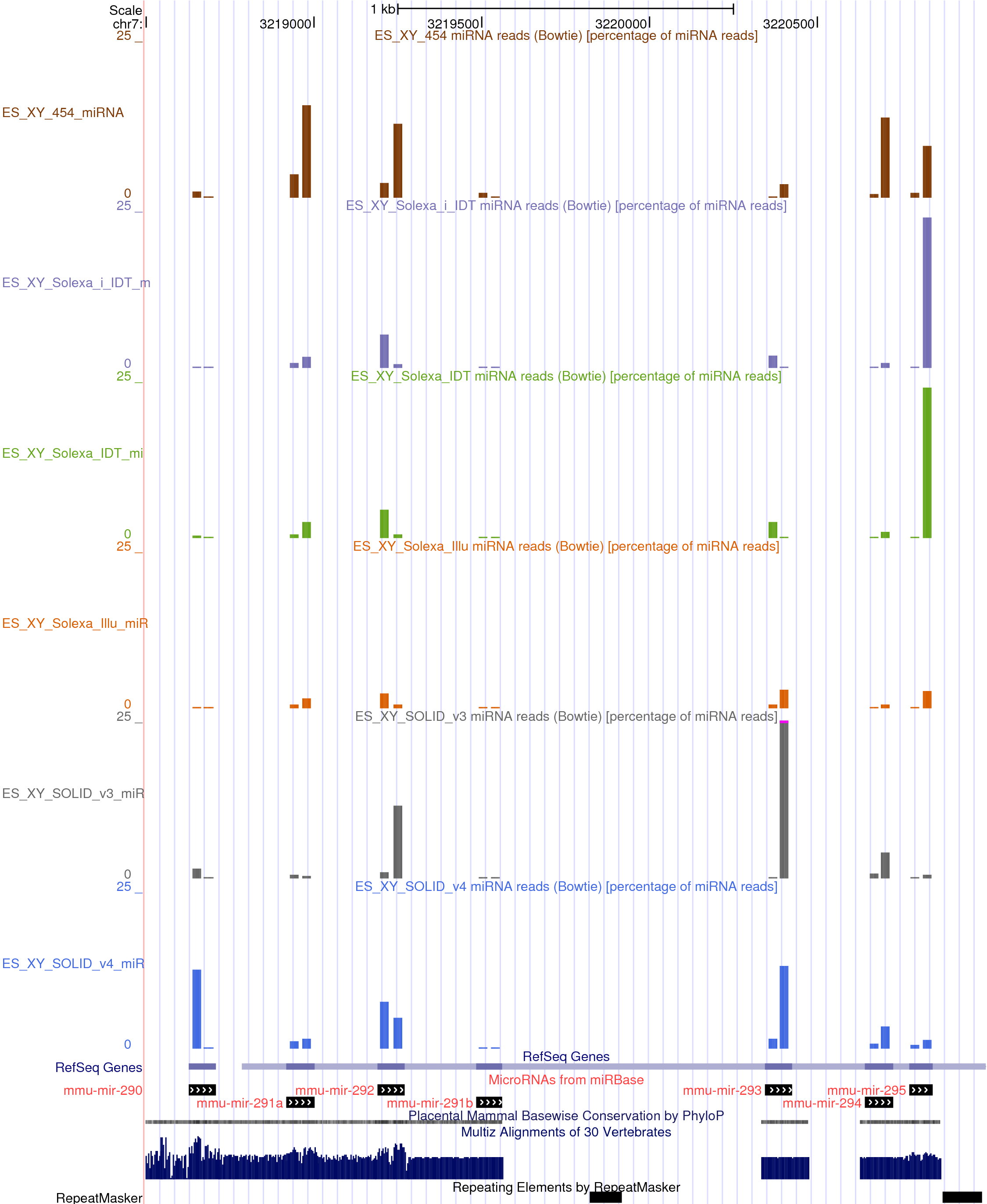

Supplement: Figure S7 — UCSC genome browser view of miR-290 cluster. UCSC genome browser screenshot showing the expression of the miR-290 cluster on chromosome 7 in the 6 XY libraries (ordered as in Table 1). In each library, the percentage for each miRNA and miR* among all miRNA-associated reads is shown. Below, the genomic positions of the pre-miRNAs, as annotated in miRBase, are shown in red. (TIFF) [file pone.0032724.s007.tif]
